# Telescopic Overdenture and Implant Supported Fixed Partial Denture: A Pragmatic Treatment Approach

**DOI:** 10.1155/2015/392397

**Published:** 2015-05-27

**Authors:** Doğu Ömür Dede, M. Cenk Durmuşlar, Onur Şahın, Ayşegül Köroğlu, Özer İşısağ

**Affiliations:** ^1^Department of Prosthodontics, Faculty of Dentistry, Bulent Ecevit University, 67600 Zonguldak, Turkey; ^2^Department of Oral and Maxillofacial Surgery, Faculty of Dentistry, Bulent Ecevit University, 67600 Zonguldak, Turkey

## Abstract

This case report presents a patient who had been rehabilitated with a telescopic overdenture and implant supported fixed partial denture (ISFPD). The treatment process was as follows: (1) fabricating telescopic crowns and overdenture prosthesis for the lower jaw and a temporary complete denture for the upper jaw, (2) using the temporary denture as diagnostic and surgical guide to optimize dental implant placement, and (3) fabricating ISFPD for the upper jaw. Using the patient's existing or temporary denture not only serves as an alternative surgical guide to calibrate the dental implant locations but also helps to finish the restoration at desired dimension, size, and anatomic form.

## 1. Introduction

Prosthetic rehabilitation of a partially edentulous patient can be established by using wide range of treatment options. Most preferred prosthetic approaches are conventional removable partial denture (RPD), teeth/implant supported over-dentures, fixed partial dentures, and implant supported fixed or partial dentures (ISFPD) [1]. Conventional removable dentures, supported by remaining teeth and alveolar tissues, have been widely used. However, the traditional retention systems such as metallic clasps, frequently used in these conventional removable dentures, impose lateral forces on remaining abutments, increase abrasive wear, and cause unaesthetic appearance [2]. These retainers are also a cause to permanent deformation that leads to loss of retention and stability of the prosthesis [3]. An overdenture is defined as a removable partial or complete denture that covers and rests on one or more remaining natural teeth, roots, and/or dental implants [4]. Telescopic crowns have been used in removable partial dentures in order to connect the remaining dentition (teeth or implants) to the denture. They may also be designated as retainers in tooth-tissue supported cases [5]. When precision attachments or telescopic crowns are selected as a retainer for a RPD, removable prosthesis will be rigidly connected to the abutment teeth. Telescopic crown retained RPDs or telescopic overdentures have better retention and stability. They also protect abutment teeth and residual alveolar ridges [6]. Telescopic overdenture prosthesis allows for easy access for oral hygiene around the abutment teeth as well as easy handling of the overdenture. The purposes of root maintenance are to prevent alveolar bone resorption, to provide better load transmission, to maintain sensory feedback, and to achieve better stability of denture with emphasis on psychological aspect. Therefore, they often offer more advantages than other types of attachments [7].

Telescopic crowns which are also known as a double crown consist of an inner (primary) telescopic coping and outer (secondary) telescopic crown [8]. Primary copings provide retention and stabilization for secondary crown and also protect the tooth from dental caries and thermal irritations. The secondary crown is engaged to the primary coping to form a telescopic unit and serves as an anchor for the remainder of the dentition [1, 8]. The retention and the stability of the telescopic denture are directly related to the number and the distribution of the abutments along the dental arch and also to the taper of the primary coping marginal walls. The taper configuration of the contacting walls generates a compressive intersurface tension. The tension should be sufficiently strong enough to sustain the prosthesis in its place. An increase in the tapering of the coping walls reduces the retention between the copings. The smaller degree of the taper provides the greater frictional retention of the retainer. The walls of the abutments with short clinical height should be kept parallel to each other or the taper of the wall should be reduced (2–5°) to improve the retention [9].

The success of implant supported restorations requires detailed treatment planning and the construction of a surgical guide [10]. Implant dentistry is a multidisciplinary concept between the prosthodontist and the surgeon. The prosthodontist arranges the surgical guides from panoramic radiograph or computed tomography and the surgeon places the implants according to these directions; thus, the implants are placed at the appropriate position on the dental arches. Surgeon can take into account the anatomic landmarks, the suitable bone regions, the aesthetic areas, and the parallelism of the implants with these guides [11–13].

Wearing a complete denture may become more difficult especially for geriatric patients due to the decreasing adaptive capacity by aging [13]. Increased residual ridge resorption, physiological intraoral changes, and the development of altered muscular patterns also obstruct the adaptation capacity for complete/partial removable dentures [1]. Dental implants have become treatment options widely used for the replacement of lost teeth [11–13]. Advantages of ISFPDs are better psychological situation, protecting the alveolar bone and mastication function and decreasing prosthesis dimension, and fixed prosthesis opportunity instead of complete denture. The range of indications in implant dentistry was expanded in the past decades from fully edentulous to partially edentulous jaws. The success of implant therapy primarily depends on appropriate treatment planning, properly performed implant surgery, and prosthetic protocol [14]. The present case reports the oral rehabilitation of a patient with telescopic overdenture and implant supported fixed partial denture. In order to increase the success rate of the present case, an appropriate treatment plan was created and surgical-prosthetic treatment procedures were performed.

## 2. Clinical Report

**Figure 1:**
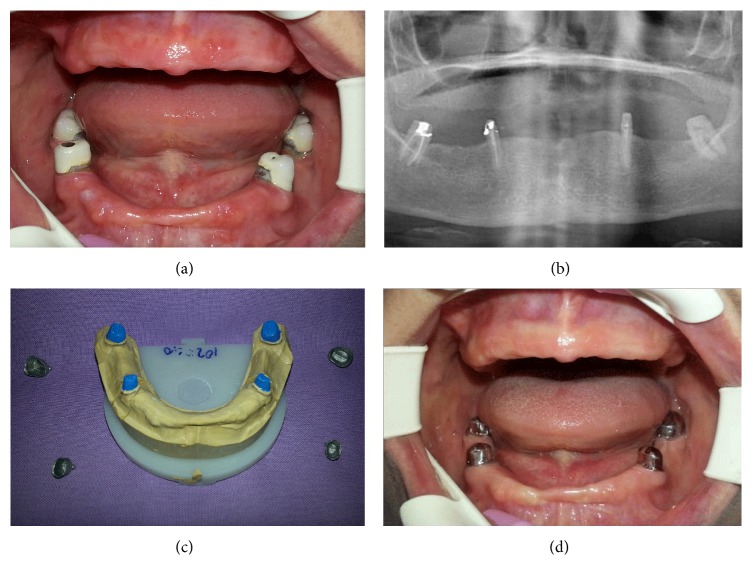
(a) Frontal and (b) orthopantomographic view of the case before treatment. The image of the primer copings on (c) cast model (d) and intraoral examination.

A 50-year-old female patient referred to Department of Prosthodontics, Faculty of Dentistry, Bulent Ecevit University, for her esthetic problems and chewing inability. After obtaining her medical, dental, and social histories, she was examined clinically and radiographically. It was determined that she had lost her all teeth in the upper jaw and many of the teeth in the lower jaw due to periodontal diseases and decays. Intraoral examination revealed that only 34, 37, 44, and 47 numbered teeth remained and all of them had crown restorations (Figure 1(a)). She had also a partial denture for lower and a complete denture for upper jaws and both prostheses had need to a renovation due to the unaesthetic appearance, base plate disturbancy, and marginal conformity problems.

The radiographs were taken to evaluate the condition of the teeth to be retained for overdenture and the condition of the bone for implants (Figure 1(b)). Patient was informed about the various treatment options like implant/tooth supported removable and implant supported fixed prostheses. When the remaining teeth/primarily periodontal tissues health and patient expectations/benefits were considered, it was decided to perform a tooth supported telescopic overdenture for mandibular arch and an ISFPD for maxillary arch. The treatment process was achieved into three parts: (1) fabricating telescopic crowns and overdenture prosthesis for the lower jaw and a temporary complete denture for the upper jaw, (2) using the temporary denture as diagnostic and surgical guide to optimize dental implant placement and placing dental implants, and (3) fabricating ISFPD for the upper jaw.

At the initial phase of first stage treatment protocol, old crowns were removed carefully without any periodontal injury. Intentional root canal and periodontal treatments were performed to the remaining teeth. Then, tooth preparations with a circumferential shoulder margin configuration were performed with tapered walls (2–5°). After abutment preparations, impressions were made with an additional polyvinyl siloxane elastomeric impression material (Elite HD, Zhermack, Italy) in a two-stage putty wash technique by using fabricated trays. The provisional restorations were fabricated for abutment teeth by using direct provisional restorative material (Luxotemp, 3M Espe, St. Paul, USA) and cemented with eugenol-free zinc oxide cement (Cavex Temp Cem, Harlem, Netherlands). The impression of the upper jaw was made with an irreversible hydrocolloid material, to fabricate the custom tray.

**Figure 2:**
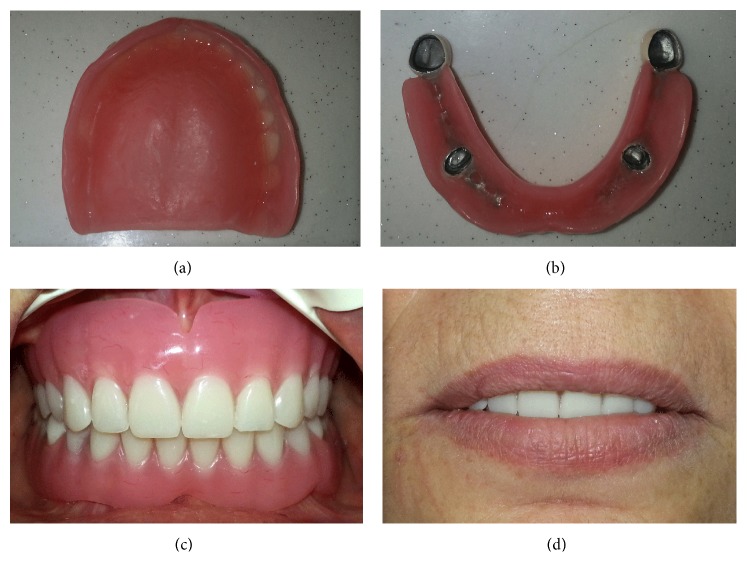
The image of the (a) temporary denture and (b) overdenture. (c) Intraoral view and (d) frontal view of the dentures.

The silicone impression was poured in a die material to obtain the cast. During the fabricating of primary copings, the cast placed on to the milling table and wax patterns of primary copings were milled in accordance with an appropriate path of insertion. Then, primary copings were casted using a nonprecious cobalt-chromium alloy and examined in the mouth (Figures 1(c) and 1(d)). After the intraoral examination, second impressions were made for both jaws with a medium body additional silicone impression material by using custom acrylic resin trays to obtain casts on which the secondary copings and base plates were fabricated. The fit of the secondary copings over the primary copings was evaluated in the patient's mouth. The frictional contact between the primary and secondary copings helped in the retention of the prosthesis. Horizontal and vertical maxilla-mandibular records were obtained with base plates and transferred to a semiadjustable articulator in order to perform the arrangement of appropriate artificial teeth. After the arrangements were evaluated intraorally according to phonetics, aesthetics, vertical dimension, and centric relations, the dentures were processed, finished, and polished (Figures 2(a) and 2(b)). After the primary copings were cemented with a zinc poly-carboxylate cement (Adhezor Carbofile, Spofa Dental, Jicín, Czech Republic), dentures were delivered to the patient (Figures 2(c) and 2(d)).

**Figure 3:**
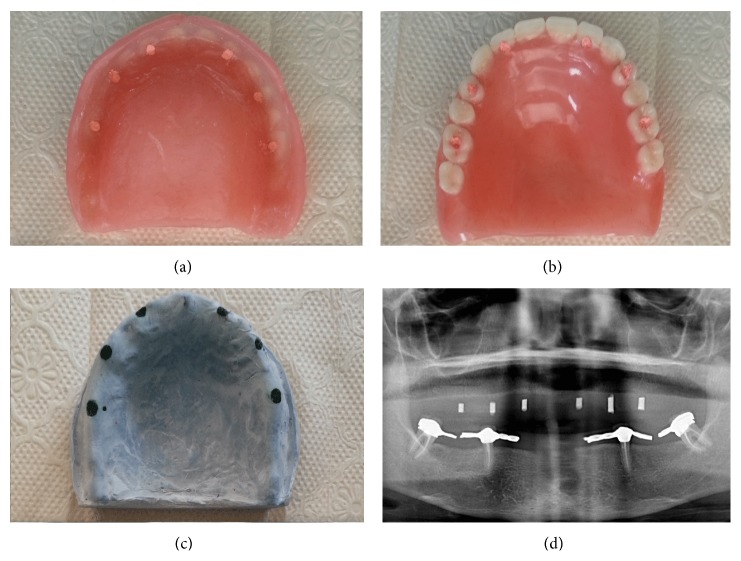
((a), (b)) The images of temporary denture with gutta percha filling material and (c) stone model that the estimated implant places were marked. (d) Orthopantomographic view of the case with gutta percha filled temporary denture.

**Figure 4:**
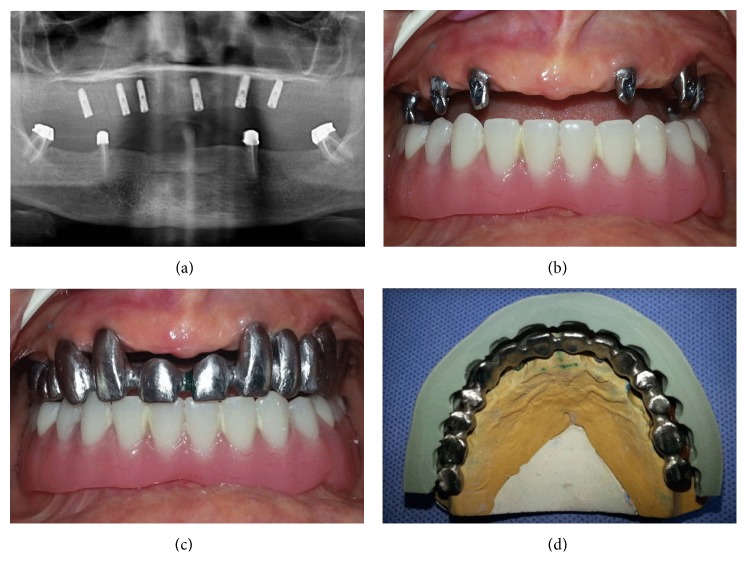
(a) Orthopantomographic view of the case with dental implants. Intraoral view of implant abutments (b) and titanium framework (c). (d) The image of titanium framework with silicone index on the stone model.

In second stage, 16, 14, 12, 22, 24, and 26 numbered teeth on maxillary temporary denture were drilled vertically and the holes filled with radiopaque gutta percha filling material (Figures 3(a)–3(c)). A panoramic radiograph was taken with this denture to assess the bone and the anatomic landmarks (Figure 3(d)). After the radiographic evaluations were performed, filling materials were removed with a heated spatula and holes cleaned by acetone and rinsed. After the local infiltrative anesthesia was performed, the temporary denture was placed into the mouth and exact position of the implant was marked on maxillary jaw by the pilot drill. Then the denture was removed and a crestal incision was made with no. 15 BP blade and handle and a full thickness flap was raised to access the alveolar bone. Because of the presence of a large fibrotic tissues and inadequate bone width at the site area of 14 numbered tooth where it was planned to place implant before surgery, the implant had to be placed into the 13 numbered tooth site area. The subsequent drills were used according to the manufacturer's specifications to the required diameter and length. Then six dental implants (BEGO GmbH, Semados Implants, Germany) were placed into 16, 13, 12, 22, 24, and 26 regions and the flap repositioned and sutures were placed (Figure 4(a)). Sutures were removed after one week from the operation and the temporary denture was adapted and relined with a soft relining material (Ufigel Soft, Voco GmbH, NY, USA) in order to be used during osseointegration period. Radiographic and clinical evaluations were performed during this period and relining material was also replaced monthly.

**Figure 5:**
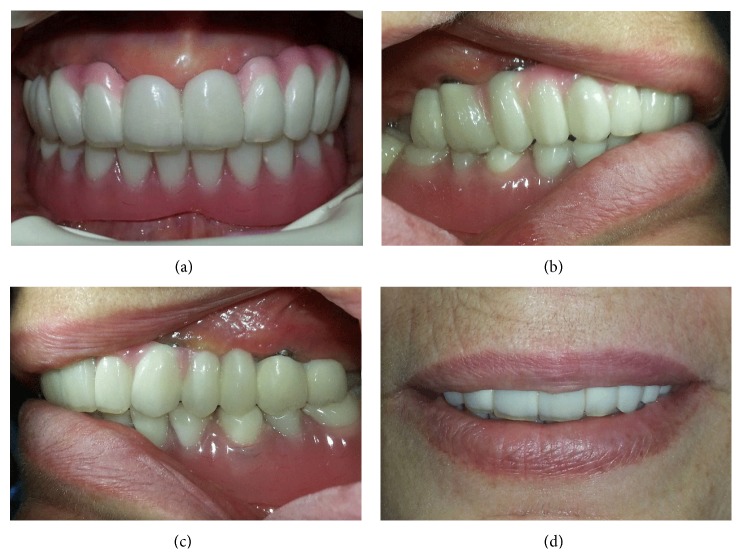
(a) Intraoral, ((b), (c)) sagittal, and (d) frontal views of the case after treatment.

After the osseointegration period of three months for maxilla, second stage surgery was performed for the maxillary implants and gingival formers were placed. In order to perform the last stage treatment protocol, two weeks later gingival formers were replaced with impression posts and impressions were made with an additional silicone impression material by using custom acrylic resin tray to obtain a master model. An impression was also made from overdenture prosthesis with an irreversible hydrocolloid material to obtain a cast for occlusal relation record. After the appropriate fabricated titanium abutments were selected and mounted (Figure 4(b)), digitizing of implant abutments in the master model was performed with a digital model scanner (3 Series, Dental Wings Inc., Montreal, Canada). A full arch fixed partial denture was digitally designed (DWOS, Dental Wings Inc., Montreal, Canada) and CAM was performed with a CNC-milling machine (D 43W, Yanadent Inc., İstanbul, Turkey) from a PMMA block. The PMMA framework analog of the restoration was evaluated intraorally and then the main framework was milled from a homogenous block of titanium grade 5 (RematitanM/Ti-4, Dentaurum GmbH & Co. KG, Ispringen, Germany). Then the marginal fit and occlusion of the main framework were also evaluated intraorally (Figure 4(c)) and the casts were transferred to a semiadjustable articulator. In order to lay the veneering material, silicone indexes were obtained from temporary denture (Figure 4(d)). A zirconium oxide reinforced composite resin material (Dialog occlusal, Schütz Dental GmbH, Rosbach, Germany) was used to complete implant supported full arch fixed partial denture, according to the manufacturer's recommendations. After the arrangements were evaluated intraorally for phonetics, aesthetics, vertical dimension, and centric relation, the dentures were finished, polished, and cemented with a zinc phosphate cement (Adhesor Fine, Spofa Dental, Jicín, Czech Republic) (Figures 5(a)–5(d)). Upper complete denture was used as surgical guide, temporary denture, and silicone index for desired prosthesis successfully. Aesthetically and functionally sound occlusion was achieved. By this way, patient satisfaction and comfort were provided.

## 3. Discussion

Total or partial edentulism not only leads to patient's impairment of oral function but also influences facial appearance and psychological conditions [1]. Rehabilitation of an edentulous patient is a complex situation that several treatment options should be developed to solve this problem. While the conventional dentures like removable partial or complete dentures are the most preferred restoration options, they also have several limitations. In removable complete or partial dentures, retention and stability are the main factors to reach the success of the rehabilitation [15]. A telescopic overdenture has advantages of good retentive and stabilizing properties, rigid splinting action, and better distribution of stresses [7]. In the current study, according to the periodontal condition, the distribution, and the number of remaining teeth, teeth supported overdenture would be the most appropriate treatment option for mandibular partial edentulism. The main advantage of the telescopic overdenture in the present case is providing balanced stress distribution between teeth-soft tissues. The telescopic retainers decrease the proportion of most traumatic lateral forces and transmit the occlusal forces in the direction of the long axis of the abutment teeth [9]. Furthermore, due to the well stress distribution and continued proprioceptive sensation, telescopic overdenture also prevents residual alveolar bone resorption [16]. It is also more aesthetic and hygienic then conventional removable partial dentures.

In order to have a successful result for implant supported restorations, precise treatment planning before surgery is necessary to avoid anatomical structures' complications and to place implants in such a way that will not affect the fabrication of esthetic restorations [11]. Surgical guide techniques are beneficial for both patient and dentist to simplify the operation and hold the desired limits and increase the sensitivity [11, 12]. In a recent study, the placement of 104 implants by 25 stereolithographic surgical guides was evaluated and 96% success rate was indicated with a 2–8 mm apical/coronal position deviation [17]. The location of implant is often critical in the success or failure of a prosthetic restoration. Although the CAD/CAM stereolithographic surgical guides, which were designed and fabricated according to computerized tomographic images, are the optimal technique, there are a lot of alternative approaches in fabricating surgical guides. In the present case report, a temporary denture was modified and used as diagnostic and surgical guide to optimize the location of dental implant placement as much as possible. This guide helps us to determine the desired implant places during panoramic radiographic images and in the beginning of surgery stage. This technique only allows for correct initial positioning and does not allow for correct inclination of implant placement. Particularly for implant supported fixed prosthesis, when the dental implant placed at undesirable locations or overangulations, hygienic problems and esthetic disharmonies may be comprisable [10].

After the placement of dental implants, the temporary denture was used not only for immediate rehabilitation during osseointegration period but also used to determine and transfer the patient's vertical-horizontal relationship. Additionally, the final implant supported fixed prosthesis was fabricated according to the guidance of silicon mold records of the temporary denture. These records helped us to finish the ISFPD at desired dimension, size, and anatomic form and also prevented the unexpected failures. Because of the great advantages of titanium alloy such as good biocompability, lower weight, and well physical and mechanical properties, ISFPD's framework was fabricated by using grade 5 titanium block. Stress distribution feature of titanium frameworks which was already noticed in a previous study was another reason for preference [18]. In order to maintain the stress distribution for ISFPD, titanium framework was layered by zirconium oxide (ZrO_2_) reinforced indirect laboratory composite resin. Recent studies indicated similar mechanical and esthetic properties for these materials according to natural tooth structure [19, 20]. Additionally, these materials have satisfactory characteristics like more translucent and esthetic structure, advanced wear character, and better and easier polishing [19].

## 4. Conclusion

Tooth supported overdentures with telescopic crowns may be preferred in the rehabilitation of partial edentulous patients to the conventional removable dentures, because of their advantages such as better retention, stability, stable occlusion, and chewing function due to the conservation of proprioception feedback. Using the patient's existing or temporary denture as an alternative surgical guide may assist to calibrate dental implant locations. Its usage as a fabrication guide may also helped to finish the restoration at desired dimension, size, and anatomic form. Implant supported fixed partial dentures are good alternatives to the conventional complete dentures due to the better reconstruction of the patient's functional, phonation, and esthetic requirements. Using titanium and resilient composite veneer materials to fabricate ISFPD may provide low abrasive wear of opposing teeth, better stress distribution, and more absorption of functional stresses.
